# Antibiotic Prophylaxis and Surgical Site Infections in Septoplasty

**DOI:** 10.1111/coa.70145

**Published:** 2026-07-15

**Authors:** Sara Sainio, Elin Fierens, Antti Mäkitie, Markus Lilja, Morag Tolvi, Marie Lundberg

**Affiliations:** ^1^ Department of Otorhinolaryngology—Head and Neck Surgery Helsinki University Hospital and University of Helsinki Helsinki Finland; ^2^ Aalto University Espoo Finland; ^3^ Research Program in Systems Oncology, Faculty of Medicine, University of Helsinki Helsinki Finland

**Keywords:** antibiotics, postoperative infection, septal deviation, septoplasty, surgical site infection

## Abstract

**Background:**

Surgical site infection is one of the most common complications of septoplasty. The use of antibiotic prophylaxis has been debated for years. Since the practice at our centre has shifted towards a more frequent use of prophylactic antibiotics in septoplasties, our aim was to review its use and effect on the postoperative infection rate.

**Methods:**

We retrospectively reviewed the medical records of all adult patients who had undergone septoplasty at the Helsinki University Hospital (Helsinki, Finland) during 2019–2023. Patient and surgery characteristics, use of surgical antibiotic prophylaxis, surgical site infections and their management were reviewed.

**Results:**

The series consisted of 1169 septoplasties, and antibiotic prophylaxis was used in 90.1% of the cases. A postoperative infection was diagnosed in 1.9%. Longer operative time and the use of silicone splints were associated with a higher risk of postoperative infections (*p* = 0.007 and *p* = 0.03, respectively). There was no significant difference in the infection rate between those who received preoperative antibiotic prophylaxis and those who did not. Among those using a preoperative course of topical antibiotic cream, no infections were seen. Compared with previous studies on septoplasty infections at our centre, the infection rate has decreased whilst the use of antibiotic prophylaxis has increased.

**Conclusion:**

Based on the present results, antibiotic prophylaxis does not affect postoperative infection rate after septoplasty. A preoperative course of nasal mupirocin seems to be a promising and less harm‐causing form of prophylaxis. To confirm our results, there is a need for analysis of larger patient cohorts and prospective, controlled studies.

## Key Points

- There is a lack of consensus and guidelines regarding antibiotic prophylaxis in septoplasties.
- Surgical site infection after septoplasty was diagnosed in 1.9% of patients, with *Staphylococcus aureus* as the most common cultured pathogen.
- 90.1% of all patients undergoing septoplasty had some kind of surgical antibiotic prophylaxis.
- There was no statistically significant difference in infection rate between those receiving antibiotic prophylaxis and those not receiving it.
- Compared with the literature, surgical site infections after septoplasty seem to have decreased as the use of preoperative antibiotic prophylaxis has increased.

## 1 Introduction

Septoplasty is one of the most commonly performed otorhinolaryngological procedures, and surgical site infection (SSI) is one of its most common complications. A postoperative infection rate of 2.0%–12.0% has previously been reported [1, 2, 3, 4, 5, 6, 7, 8, 9]. Although these postoperative infections are mainly mild to moderate and can be fully treated with antibiotics, there are reports on more serious infections, such as toxic shock syndrome and endocarditis [10, 11]. Diabetes and concurrent turbinoplasty further increase the risk for SSI after septoplasty [1, 6, 12].

For years, the use of surgical antibiotic prophylaxis in septoplasty has been studied and discussed. Nevertheless, a lack of consensus and specific guidelines regarding possible antibiotic prophylaxis in septoplasty remains. Generally, surgical antibiotic prophylaxis is recommended in clean‐contaminated and contaminated surgical procedures, thus also including septoplasties [13]. We have previously found preoperative single‐dose antibiotic prophylaxis to significantly decrease the risk of SSI [2, 3]. Zavdy et al. recently reported similar results [1]. In the prospective, randomised study by Lilja et al., published in 2011, there was no statistical difference in postoperative infection between the group receiving antibiotic prophylaxis and the group not receiving it. However, there was a trend towards a higher SSI risk without prophylaxis [7]. In contrast, antibiotic prophylaxis in septoplasty has been found redundant by Dąbrowska‐Bień, Mäkitie et al., Ricci et al. [6, 9, 14]. Additionally, a recent meta‐analysis by Benites et al. examined antibiotic prophylaxis in septoplasty, septorhinoplasty and rhinoplasty, and found no significant difference in the SSI rate between the patients receiving prophylaxis and those not [15].

Based on our previous results, the current septoplasty protocol at our centre since 2019 includes one dose of intravenous antibiotic (primarily 1500 mg cefuroxime, alternatively 600 mg clindamycin) prophylaxis, administered 30–60 min preoperatively for all septoplasty patients. Therefore, our aim was to evaluate quality assurance regarding this guideline, as well as its effect on postoperative infection rate, and how these results compare with our previous reports.

## 2 Methods

We retrospectively reviewed the medical records of all patients aged 16 years and older who had undergone septoplasty or septocolumelloplasty (NOMESCO code DJD20) at the Helsinki University Hospital (Helsinki, Finland) during 2019–2023. Surgeries, in which septoplasty was combined with another procedure that was evaluated to increase the risk for a postoperative infection, were excluded (i.e., endoscopic sinus surgery, dacryocystorhinostomy, rhinoplasty). A turbinoplasty with either cold instruments or radiofrequency was not excluded. The recorded parameters included patient characteristics, closure methods, use of prophylactic antibiotics, follow‐up and postoperative infections.

### 2.1 Surgical Procedure

Surgeries were carried out under either local (topical cocaine cum adrenalin and infiltrated lidocaine cum adrenalin) or general anaesthesia. General anaesthesia was used if the patient requested it or if the surgeon deemed it necessary. Surgical methods were chosen by the surgeon based on septal pathology. Generally, methods like swinging door or re‐insertion of extracorporeally straightened cartilage were used. Antibiotic prophylaxis, chosen by the surgeon, was administered as topical nasal mupirocin/fusidic acid for 5–7 days preoperatively, intravenous cefuroxime (or other intravenous antibiotic in case of allergy) 30–60 min preoperatively or oral cephalexin (or other oral antibiotic in case of allergy) 7 days postoperatively. If nasal packing was used, it was removed the first postoperative morning. Silicone splints were removed approximately 7 days postoperatively. The surgeon planned the postoperative follow‐up individually. Patients who were not scheduled for routine follow‐up were advised to contact the hospital with a low threshold in case of any postoperative issues.

### 2.2 SSIs

All patients with medical records suggestive of SSI within 30 days postoperatively were reviewed carefully by one author (S.S.), and infections were identified according to the CDC criteria [16]. Cases in which an infection was possible but not certain were re‐reviewed by two other authors (M. Lundberg and M. Lilja). Disagreement was resolved by discussion until consensus was reached. Superficial SSI was defined as infection of skin/mucosa around the incision and purulent drainage, positive bacterial culture or reopened incision. Deep SSI was defined as an infection involving deep soft tissues and purulent discharge, reopened incision with positive bacterial culture and local signs of infection or fever, or abscess formation [16].

### 2.3 Ethical Considerations

The study had a research permission of the Helsinki University Hospital (HUS/15/2024). As the study was based on medical records with no patient intervention, no approval from a research ethics committee was required according to the Medical Research Act of Finland (488/1999).

This study was a non‐interventional retrospective analysis of previously collected patient data. All data were anonymised before analysis. Hence, informed patient consent was not required.

### 2.4 Statistics

The data were analysed using SPSS (version 29.0, IBM Corp. Armonk, NY, USA), applying the functions Cross Tabulation and Mann–Whitney *U*. A *p*‐value < 0.05 was considered statistically significant.

## 3 Results

During the study period there were 1738 patients who had undergone septoplasty. After reviewing the series in accordance with the exclusion criteria, 1169 patients were included. Of these patients, 303 (25.9%) were women, and the median age was 38 years (IQR 29–51). Patients with and without SSI did not differ in regards of patient characteristics, except for women having more infections than men (*p* = 0.009) (Table 1). Surgery was performed under local anaesthesia in 57.7%. When comparing the sexes, we found smoking to be less common among women (14.3% vs. 22.1%, *p* = 0.01) and general anaesthesia to be used more frequently in women (48.8% vs. 40.1%, *p* = 0.01). Altogether, surgeries were performed by 66 different surgeons. Four patients had a bacterial culture taken from their nose within 30 days preoperatively due to crusty mucosa either at a preoperative visit or on the surgery day. All of those showed *Staphylococcus aureus* , all had antibiotic prophylaxis, and none had an SSI.

**TABLE 1:** Patient and operative characteristics of septoplasty patients with and without postoperative infection.

	No postoperative infection, *N* (%)	Postoperative infection, *N* (%)	*p*
Women	292 (96.4)	11 (3.6)	0.009
Men	855 (98.7)	11 (1.3)	
Median (IQR) age, years	38 (29–51)	47 (30–55)	0.19
Current smoker			
Yes	232 (99.1)	2 (0.9)	0.41
No	911 (97.9)	20 (2.1)	
Unknown	4 (100)	0	
Diabetes mellitus			
Yes	40 (97.6)	1 (2.4)	0.79
No	1107 (98.1)	21 (1.9)	
Local anaesthesia	664 (98.5)	10 (1.5)	0.24
General anaesthesia	483 (97.6)	12 (2.4)	
Surgery by			
Rhinosurgeon	385 (97.5)	10 (2.5)	0.45
ENT surgeon	586 (98.3)	10 (1.7)	
Resident	176 (98.9)	2 (1.1)	
Revision septoplasty			
Yes	78 (97.5)	2 (2.5)	0.87
No	1068 (98.2)	20 (1.8)	
Median (IQR) surgery duration, minutes	76 (57–100)	100 (73–124)	0.007
Postoperative packing			
Yes	504 (98.8)	6 (1.2)	0.12
No	643 (97.6)	16 (2.4)	
Postoperative splints			
Yes	665 (97.4)	18 (2.6)	0.03
No	482 (99.2)	4 (0.8)	
Stapler			
Yes	311 (99.0)	3 (1.0)	0.16
No	836 (97.8)	19 (2.2)	
Concurrent turbinoplasty			
Yes	351 (98.0)	7 (2.0)	0.90
No	796 (98.2)	15 (1.8)	

### 3.1 Surgical Antibiotic Prophylaxis

The majority (1053 patients, 90.1%) had some kind of surgical antibiotic prophylaxis (Table 2). Of these, 953 had preoperative systemic antibiotics, most often cefuroxime 1500 mg intravenously 30–60 min before incision. In cases of allergy, another antibiotic was used instead of cefuroxime (clindamycin, *n* = 10; ciprofloxacin because of amoxicillin and clindamycin allergy, *n* = 1). Levofloxacin was used in one case of a preoperative MRSA culture. A preoperative course of topical mupirocin/fusidic acid cream was used alone by 12 patients, and in combination with a preoperative systemic antibiotic by 95. Postoperative antibiotics, most commonly a 1‐week course of cephalexin, were used alone by 90 patients, and in combination with preoperative systemic antibiotics by 50.

**TABLE 2:** Number of surgical site infections among septoplasty patients receiving antibiotic prophylaxis versus not receiving it.

	No infection, *N* (% of total)	Infection, *N* (% of total)	*p*	OR (95% CI)
Any antibiotic prophylaxis	1034 (98.2)	19 (1.8)	0.56	1.4 (0.4–5.0)
Preoperative systemic antibiotics	934 (98.1)	19 (1.9)	0.67	1.3 (0.4–4.5)
Preoperative topical antibiotics	119 (100)	0	0.08	N.A.
Preoperative systemic and topical antibiotics	107 (100)	0	0.09	N.A.
Postoperative antibiotics	140 (100)	0	0.06	N.A.
Preoperative and postoperative antibiotics	50 (100)	0	0.25	N.A.
No antibiotic prophylaxis	113 (97.4)	3 (2.6)		

### 3.2 SSIs

An SSI was diagnosed in 22 patients (1.9%, Table 2). All infections, except one, were treated with oral antibiotics. One patient was treated overnight at the hospital with intravenous cefuroxime and metronidazole followed by oral cephalexin at home. One patient was treated with oral clindamycin and nasal fusidic acid, and one patient with nasal mupirocin alone. The rest (*n* = 19) were treated with cephalexin; three of these had nasal mupirocin added to the treatment. No SSI was treated surgically, but three had a mucosal incision performed at the emergency room. All of these were classified as deep infections, with two of them diagnosed with a septal abscess.

Of those diagnosed with a postoperative infection, 13 had bacterial cultures taken, of which one remained negative. In the rest, the bacteria cultured were *S. aureus* (*n* = 4), multiple pathogens including *S. aureus* (*n* = 4), and *Citrobacter*, *Escherichia coli* , MRSA, *Serratia marcenscens* (*n* = 1 each). Nineteen of the postoperative infections were evaluated as superficial, whereas three were deep infections. Of those with deep infections, bacterial cultures showed MRSA, *S. aureus* and *S. marcenscens* . Two had received preoperative cefuroxime, and one had no antibiotic prophylaxis.

Notably, 37 patients (3.2%) had a mention of poor condition of the nasal mucosa (i.e., dryness, crusts, large mucosal perforation) preoperatively. Two of these had an SSI, with no significant association between poor mucosa and SSI (*p* = 0.47). All but three patients with poor mucosa had antibiotic prophylaxis. Of the three that did not have antibiotic prophylaxis, none had SSI.

The use of silicone splints was associated with SSI (*p* = 0.03), as was a longer surgery duration (*p* = 0.007). We did not find any significant association between the use of preoperative antibiotic prophylaxis and SSI (Table 2). None of the patients who received topical antibiotic cream preoperatively had an SSI. In multifactorial analysis, this result was strengthened (data not shown).

### 3.3 Postoperative Follow‐Up

A majority of 811 patients (69.4%) had a follow‐up visit within 30 days postoperatively. Additionally, seven (4.1%) had a follow‐up contact by phone. Of the follow‐up visits, 790 (97.4%) were scheduled, 11 (1.4%) were visits to the emergency room and 10 (1.2%) urgent visits scheduled based on the patients contacting the hospital. Patients not scheduled for routine follow‐up were advised to maintain a low threshold for contacting the hospital postoperatively in case of any abnormal postoperative symptoms.

## 4 Discussion

The postoperative septoplasty infection rate of 1.9% in this series of 1169 patients is comparable to that of previous literature [1, 2, 3, 4, 5, 6, 7, 8, 9]. At our department, however, the current infection rate is lower than in the previous reports by our centre and shows an inverse trend when comparing to the use of preoperative prophylactic antibiotics [2, 3, 5, 7, 8, 9]. The decrease in infection rate might, of course, be influenced by changes in patient‐related factors, technique, as well as postoperative protocols. In comparison with those of previous studies, however, there were no significant changes in age or sex distribution or smoking habits. Surgical technique and preoperative washing have remained largely the same over the past 20 years. Compared with earlier studies, the use of packing has decreased whereas the use of splints has increased, with current rates being comparable to those of Lundberg et al. [2]. In addition, the use of local anaesthesia has steadily decreased over the years. Considering our results showing association between splints as well as a tendency of infections occurring more frequently after surgeries performed under general anaesthesia, these changes in surgical practices seem unlikely to explain the decrease in infection rate.

Interestingly, we found that use of silicone splints was associated with a higher infection rate. Silicone splints are a foreign body and hence serve as a possible growing ground for bacteria and biofilm, as demonstrated by Ritter et al., who found 93% of postoperative bacterial cultures from silicone splints to be positive [4]. Despite this, no difference in infection rate was found in a large meta‐analysis comparing splints to no splints [17]. Considering our results, however, a reduction in SSIs might be achieved by limiting the use of silicone splints to cases where prevention of synechiae is of special importance.

In addition to the use of silicone splints, we found a longer surgery duration to be associated with a higher infection rate. A similar tendency was found by Lilja et al. [7]. Of course, no surgeon prolongs surgery more than necessary, but the increased infection risk with longer surgeries is worth acknowledging. In 2022, Lundberg et al. showed a higher infection rate among patients operated on under general anaesthesia [2]. A similar tendency could be seen in our material. This may be explained by more complicated surgeries tending to be performed under general anaesthesia and requiring longer surgery duration. In contrast to previous literature, we did not find diabetes or concurrent turbinoplasty to affect the infection rate [1, 6, 12]. We did not find a preoperative mention of poor mucosa to associate with SSI. However, the majority of those with poor mucosal conditions preoperatively did receive antibiotic prophylaxis, which might affect the results.

Interestingly, SSIs were more common in women than in men—a result not found in previous literature. This could be explained by biological factors, such as local mucosal inflammation due to hormonal rhinitis as well as differences in immune response between the sexes. As women were operated on under general anaesthesia more often than men, it is also possible that the chosen method of anaesthesia affects this difference between women and men.

Even though bacterial cultures were obtained in only 59% of SSIs, the microbiology of those was similar to that of previous studies, with *S. aureus* being the most common pathogen [7]. In future studies as well as when treating SSIs, it is of essence to obtain bacterial cultures to allow direction of antimicrobial therapy specifically against the causative pathogen.

The evolution of antibiotic resistance and effect of antibiotics on gut microbiome are well known, and thus, the need for antibiotics should always be carefully considered. We could not find a significant difference between patients receiving one dose of preoperative systemic antibiotics and those who did not. However, compared with Lundberg et al., as the use of preoperative antibiotic prophylaxis has increased (90% vs. 61%), the infection rate has decreased (1.9% vs. 3.8%) [2]. Interestingly, no patient with a postoperative 1‐week course of oral antibiotics had a postoperative infection. It is possible that these results are affected by the comparably low number of patients with postoperative prophylaxis. Considering the effect on gut microbiome, the risk of developing antibiotic resistance, and the risk for adverse effects associated with longer courses of antibiotics, a one‐dose prophylaxis could be more justifiable than a 1‐week course of antibiotics and is also recommended by WHO [18].

Few patients in our material used topical nasal antibiotic cream but none of them had an SSI. The use of topical antibiotics as antibiotic prophylaxis in septoplasty has not previously been studied. However, soaking nasal packings in these antibiotic creams was found to decrease postoperative nasal bacterial growth [19]. In their guidelines for prevention of SSIs, the World Health Organization also suggests using perioperative topical nasal mupirocin for patients with known nasal colonisation of *S. aureus* [18]. Previous studies have shown that 35%–46% of septoplasty patients do have a preoperative *S. aureus* colonisation [7, 20]. Hence, it would be of interest to study how a more widespread use of preoperative mupirocin/fusidic acid affects the infection rate.

This is, to our knowledge, the largest patient cohort focusing specifically on postoperative infections after septoplasty. Despite some variance in the execution of antibiotic prophylaxis and follow‐up, the large and unselected patient material in a real‐world setting is the strength of the study. Even though SSIs after septoplasty are rarely life‐threatening, an infection can ruin surgical results and, in addition, causes personal harm to the patient and increased costs through increased need for healthcare services and sick leave. As a result, continued work for further decreasing the postoperative infection rate after septoplasties is needed.

## 5 Conclusion

Postoperative infections after septoplasty are rare. Use of silicone splints and a longer surgery duration associates with postoperative infections. Antibiotic prophylaxis was not found to affect the infection rate in the current study. However, compared with previous studies, the infection rate seems to decrease with increased use of antibiotic prophylaxis. The ideal execution of antibiotic prophylaxis remains unclear, and thus, further, ideally prospective studies are warranted, possibly comparing a course of preoperative topical antibiotics with one‐dose preoperative systemic antibiotics.

## Author Contributions

**Sara Sainio:** conceptualisation, formal analysis, investigation, visualisation, writing – original draft preparation. **Elin Fierens:** investigation, writing – review and editing. **Antti Mäkitie:** conceptualisation, writing – review and editing. **Markus Lilja:** conceptualisation, writing – review and editing. **Morag Tolvi:** conceptualisation, writing – review and editing. **Marie Lundberg:** conceptualisation, project administration, writing – review and editing.

## Funding

This work was supported by Helsingin ja Uudenmaan Sairaanhoitopiiri.

## Consent

This study was a non‐interventional retrospective analysis of previously collected patient data. All data were anonymized before analysis. Hence, informed patient consent was not required.

## Conflicts of Interest

The authors declare no conflicts of interest.

## Data Availability

The data that support the findings of this study are available from the corresponding author upon reasonable request.
